# Childhood cancers in a section of the South African private health sector: Analysis of medicines claims data

**DOI:** 10.4102/hsag.v25i0.1382

**Published:** 2020-09-30

**Authors:** Marianne N. Otoo, Martie S. Lubbe, Hanlie Steyn, Johanita R. Burger

**Affiliations:** 1 Medicine Usage in South Africa, Faculty of Health Sciences, North-West University, Potchefstroom, South Africa

**Keywords:** epidemiology, childhood cancer, adolescent, incidence, incidence trends, private health sector, South Africa

## Abstract

**Background:**

Although childhood cancers are rare, increases in incidence have been observed in recent times. There is a paucity of data on the current incidence of childhood cancers in South Africa.

**Aim:**

This study described the epidemiology of childhood cancers in a section of the private health sector of South Africa, using medicines claims data.

**Setting:**

This study was designed on a nationally representative medicine claims database.

**Method:**

A longitudinal open-cohort study employing children younger than 19 years and diagnosed with cancers between 2008 and 2017 was conducted using medicine claims data from a South African Pharmaceutical Benefit Management company. Cases were identified using International Classification of Diseases, Tenth Revision (ICD-10) diagnostic codes C00 to C97, together with a medicine claim reimbursed from oncology benefits. Crude incidence rates were calculated per million persons younger than 19 years on the database and standardised using the Segi 1960 world population. Temporal trends in incidence rates, analysed using the joinpoint regression, were reported as annual percentage changes (APCs).

**Results:**

Overall, 173 new cases of childhood cancers were identified in the database, translating into an age-standardised incidence rate (ASR) of 82.3 per million. Annual incidence of cancer decreased from 76.7 per million in 2008 to 58.2 per million in 2017. More incident cases were identified in males (68.8%). The highest proportion of incident cases was recorded for leukaemias (39.9%), the 5–9 year age group (34.1%) and the Gauteng Province (49.7%).

**Conclusion:**

The incidence of childhood cancers decreased over time in the section of the private health sector studied. Leukaemias were the major drivers of childhood cancer incidence.

## Introduction

Childhood cancers, which are described as a varied collection of malignancies, are uncommon and form only a small proportion of all cancers (Erdmann et al. 2015:2628; Israels et al. 2015:607; Murphy et al. 2013:95; Yang et al. 2014:285). There are, however, approximately 300 000 newly diagnosed cases of cancers annually in children aged 0–19 years globally (Lam et al. 2019:1182), with over 80% of these cases occurring in resource-limited countries (Magrath et al. 2013:104; White, Castle & Haig 2013:1091). Notwithstanding the rarity of childhood cancers, increases in incidence have been observed in recent times in some parts of the globe (Bidwell et al. 2019; Sommer et al. 2019:159; Steliarova-Foucher et al. 2017b:727). A study using data from 153 registries in 62 countries across the globe indicated an increase in the incidence of childhood cancers from 124.0 cases per million person-years in the 1980s to 140.6 cases per million person-years in 2010, in children younger than 15 years (Steliarova-Foucher et al. 2017b:727).

Treatment of infectious diseases such as human immunodeficiency virus (HIV) infection, tuberculosis and malaria takes precedence in most developing countries and this has led to childhood cancers not regarded as a high priority public health concern (Elhassan et al. 2018; Slone et al. 2018:1; Stefan et al. 2017). The paucity of data for comparative studies, which could inform public health decisions, could also contribute to childhood cancers not perceived to be a high priority public health concern (Lam et al. 2019:1182; Stefan 2015a:35).

The rarity of childhood cancers makes the estimation of their occurrence reliable only if a large population of children is studied (Moreno et al. 2013:466; Steliarova-Foucher et al. 2015:1065). Data for the estimation of childhood cancer incidence are usually obtained from population- or hospital-based registries (Howard et al. 2008:463; Steliarova-Foucher 2019:460). The incidence of childhood cancers is not adequately described in low- and middle-income countries (LMIC), especially those in sub-Saharan Africa, because of a lack of high-quality data (Erdmann et al. 2018:22; Magrath et al. 2013:107). This makes the true variation of childhood cancer incidence on the continent largely unknown (Stefan 2015b:166). This is in contrast with the adequately described incidence rates in developed countries (Baade et al. 2010:622–623; Isaevska et al. 2017; Peris-Bonet et al. 2010:iii106; Siegel et al. 2018; Xie, Onysko & Morrison 2018:81–82; Zheng et al. 2015:117).

Some epidemiological studies of childhood cancer in South Africa have utilised data from the South African Childhood Tumour Registry (SACTR) (Stefan et al. 2015:940) and the National Cancer Registry (NCR) (Erdmann et al. 2015:2629), although the latter registry is not child-specific (Stefan 2010:318). The NCR has also had inconsistencies regarding data on childhood cancer, resulting in a statistic that is not up-to-date (Stefan & Stones 2012:605). The most recent data used in epidemiological studies of childhood cancer in South Africa are up to 2012 (Steliarova-Foucher et al. 2017a). Most epidemiological studies of childhood cancers in South Africa have also not described the changing patterns of childhood cancers. Current epidemiological studies are required for any cancer control strategy, to monitor changes in disease burden, and to assess the effectiveness of implemented policies (Bhakta et al. 2019:e42; Segbefia et al. 2013:65; Steliarova-Foucher 2019:460; Ward et al. 2019:483). There is, however, a paucity of information on the current epidemiological trends of childhood cancer in South Africa as a whole and the private health sector, specifically. We therefore aimed at determining the incidence and temporal trends in the incidence of childhood cancers in a section of the private health sector of South Africa by analysing medicine claims data over 10 years.

## Materials and methods

### Study design and data source

This study followed a longitudinal open cohort study design. Medicine claims data for 10 years from 01 January 2008 to 31 December 2017 were obtained from the database of a South African Pharmaceutical Benefit Management (PBM) company. The database, which is an electronic claims processing system used for managing medical schemes’ medicine benefits, acts as an interface between the medical insurance scheme and the service providers. This PBM company is one of the largest electronic claims processing and medicine benefit management companies for medical aid schemes in South Africa and covers over 1.8 million beneficiaries.

Data fields on the database that were used for this study included gender, date of birth, encrypted patient number, the date prescription was dispensed, dispensed active substances, International Classification of Diseases, Tenth Revision (ICD-10) diagnostic codes, reimbursement category and the postal code of prescriber. Prescribers’ postal codes were used as a proxy measure for the geographic location to identify where the patient received treatment. Using the unique encrypted patient number, all patients aged younger than 19 years and claiming medications from 01 January 2008 to 31 December 2017 were identified on the database and used as the denominator to estimate incidence rates of childhood cancer. The study population was then identified using the ICD-10 codes C00 to C97 recorded on medicine claims.

### Study population

The study population comprised children younger than 19 years at the time of diagnosis, stratified by age at last birthday into age groups of < 1, 1–4, 5–9, 10–14 and 15 < 19 years. Cases were identified based on ICD-10 codes C00 to C97 on claims for medicine(s) reimbursed from patients’ oncology benefits, and were categorised into the 12 main diagnostic groups of childhood cancers (Steliarova-Foucher et al. 2005:1458).

Denominator data for the estimation of incidence rates were obtained from the database of the PBM company and consisted of the total number of patients under the age of 19 years, annually from 2008 to 2017. A total of 178 564 patients younger than 19 years were obtained for 2008 compared to 249 843 for 2009, 223 267 for 2010, 195 953 for 2011, 179 901 for 2012, 188 568 for 2013, 201 723 for 2014, 215 245 for 2015, 220 066 for 2016 and 240 766 for 2017. These were further categorised by age groups (< 1, 1–4, 5–9, 10–14 and 15 < 19 years), gender and geographic location.

### Definition of geographical location

Prescribers’ postal codes were used to identify municipalities, which were then categorised into the nine provinces of South Africa and used as a proxy for geographic location because the residential address of patients is not indicated on the database. The nine provinces of South Africa include the Eastern Cape, Free State, Gauteng, KwaZulu-Natal, Limpopo, Mpumalanga, Northern Cape, North West and Western Cape, as demarcated by the Municipal Demarcation Board in 2000 (Municipal Demarcation Board 2000).

### Statistical analysis

Data were extracted and analysed using SAS^®^ version 9.4 (SAS Institute Inc. 2002–2012). Frequencies, gender ratios and incidence rates were used to describe the study population. Age-specific and overall crude incidence rates were expressed per million persons younger than 19 years on the database. Age-standardised incidence rates (ASRs), also expressed per million persons, were estimated as the weighted average of the age-specific incidence rates of the 0–4, 5–9, 10–14 and 15 < 19 year age groups, using the corresponding weights of the Segi 1960 World Standard Population (Ahmad et al. 2001). Temporal trends in the incidence of all cancers, and leukaemia separately, were estimated using the Joinpoint Regression Program (National Cancer Institute 2019) and reported as annual percentage changes (APCs) with their corresponding confidence intervals (CIs).

## Ethical considerations

Ethical approval was obtained from an authorised, licensed Health Research Ethics Committee (HREC) (ethical approval number: NWU-00179-14-A1-08). The requirement for informed consent from patients whose data were used in this study was waived by the HREC because of the retrospective design of the study and the use of administrative data. Permission was also granted by the PBM company for the use of the data from their database.

Data used for this study were anonymised by the PBM company by removing all identifying information on medical schemes, beneficiaries and service providers before being released for analyses. The dataset received as a comma-separated values file from the PBM was stored on the password-protected personal computer of the research team leader and statistician for analysis. Access to the data was subject to the signage of a confidentiality agreement.

## Results

### Childhood cancer incident cases

A total of 173 incident cases of childhood cancers were identified during the 10 years, representing 0.01% of children aged below 19 years on the database (average annual population of children younger than 19 years = 209 390). Table 1 depicts the characteristics of the study population. There were more incident cases in males (68.8%) compared with females, resulting in a male-to-female (M/F) ratio of 2.2. The mean age of the study population at diagnosis was 10.0 ± 5.4 years. The highest proportion of incident cases (34.1%) was identified in the 5–9 year age group compared to 0.6% among patients < 1 year. Leukaemias were the most frequently identified cancers (39.9%), followed by lymphomas (13.9%) and central nervous system (CNS) neoplasms (11.0%). The highest proportion of incident cases was identified in Gauteng (49.7%), followed by KwaZulu-Natal (29.5%) and the Western Cape Province (13.3%).

**TABLE 1:** Incident case counts and case frequencies from 2008 to 2017 stratified by age, gender, geographic area and malignancy type.

Subgroup	Case counts	%
**Gender**		
Male	119	68.8
Female	54	31.2
**Age groups (years)**		
< 1	1	0.6
1–4	26	15.0
5–9	59	34.1
10–14	40	23.1
15 < 19	47	27.2
**Province**		
Eastern Cape	0	0.0
Free State	8	4.6
Gauteng	86	49.7
KwaZulu-Natal	51	29.5
Limpopo	2	1.2
Mpumalanga	0	0.0
North West	2	1.2
Northern Cape	0	0.0
Western Cape	23	13.3
Not indicated	1	0.6
**Malignancy type**		
Leukaemias	69	39.9
Lymphomas	24	13.9
CNS neoplasms	19	11.0
Neuroblastoma	3	1.7
Retinoblastoma	6	3.5
Renal tumours	7	4.0
Hepatic tumours	1	0.6
Bone tumours	12	6.9
Soft tissue sarcomas	7	4.0
Germ cell tumours	6	3.5
Carcinoma and melanomas	17	9.8
Other and unspecified neoplasms	2	1.2
**Total**	**173**	**100.0**

CNS, central nervous system.

### Childhood cancer incidence rates

The age-standardised incidence rate for all cancers was estimated at 82.3 per million persons based on the 173 cases identified over the 10 years (Table 2). Overall, the highest age-specific incidence rate was estimated for adolescents aged 15 < 19 years (112.8 cases per million persons), followed by children aged 5–9 years (101.7 cases per million persons). The lowest age-specific incidence rate was estimated in children who were diagnosed at < 1 year (13.2 cases per million persons). Leukaemias, lymphomas and CNS neoplasms were the most frequently occurring childhood cancers, with ASRs of 32.6, 11.7 and 9.1 cases per million persons.

**TABLE 2:** Childhood cancers on the database from 2008 to 2017 stratified by malignancy type, age-specific, crude and age-standardised incidence rates.

Malignancy type†	Number of cases in age group (in years)					*n*	%	Age-specific incidence rates‡					Crude incidence§		ASR¶	
	< 1	1–4	5–9	10–14	15 < 19			< 1	1–4	5–9	10–14	15 < 19	0–14	0 < 19	0–14	0 < 19
Leukaemias	1	10	26	15	17	69	39.9	13.2	19.3	44.8	29.6	40.8	31.0	33.0	30.2	32.6
Lymphomas	0	1	6	6	11	24	13.9	0.0	1.9	10.3	11.9	26.4	7.8	11.5	7.4	11.7
CNS neoplasms	0	5	6	4	4	19	11.0	0.0	9.7	10.3	7.9	9.6	8.9	9.1	8.9	9.1
Neuroblastoma	0	2	1	0	0	3	1.7	0.0	3.9	1.7	0.0	0.0	1.8	1.4	1.9	1.4
Retinoblastoma	0	3	3	0	0	6	3.5	0.0	5.8	5.2	0.0	0.0	3.6	2.9	3.7	2.8
Renal tumours	0	1	6	0	0	7	4.0	0.0	1.9	10.3	0.0	0.0	4.2	3.3	4.0	3.1
Hepatic tumours	0	0	0	1	0	1	0.6	0.0	0.0	0.0	2.0	0.0	0.6	0.5	0.6	0.5
Bone tumours	0	0	1	4	7	12	6.9	0.0	0.0	1.7	7.9	16.8	3.0	5.7	2.8	6.0
Soft tissue sarcomas	0	0	3	2	2	7	4.0	0.0	0.0	5.2	4.0	4.8	3.0	3.3	2.8	3.3
Germ cell tumours	0	0	1	2	3	6	3.5	0.0	0.0	1.7	4.0	7.2	1.8	2.9	1.7	2.9
Carcinoma and melanomas	0	4	5	6	2	17	9.8	0.0	7.8	8.6	11.9	4.8	8.9	8.1	8.9	7.9
Other and unspecified neoplasms	0	0	1	0	1	2	1.2	0.0	0.0	1.7	0.0	2.4	0.6	1.0	0.5	1.0
**Total**	**1**	**26**	**59**	**40**	**47**	**173**	**100.0**	**13.2**	**50.4**	**101.7**	**79.1**	**112.8**	**75.1**	**82.6**	**73.4**	**82.3**

ASR, age-standardised incidence rate; CNS, central nervous system.

† , Childhood cancers categorised using the International Classification of Childhood Cancers, Third Edition (ICCC-3).

‡ , Age-specific incidence rates expressed per 1 000 000 persons younger than 19 years.

§ , Crude rates expressed per 1 000 000 persons aged 0–14 years and all patients younger than 19 years.

¶ , Age-standardised incidence rate expressed per 1 000 000 persons aged 0–14 years and all patients younger than 19 years.

The age-standardised incidence rate for children younger than 15 years was 73.4 cases per million persons. Leukaemias, CNS neoplasms, and carcinomas and melanomas constituted the three most frequently diagnosed cancers in this age group, with ASRs of 30.2, 8.9 and 8.9 cases per million persons, respectively (Table 2).

A higher overall ASR was estimated for males (112.3 cases per million persons) compared with females (51.9 cases per million persons) (Table 3). Leukaemias, lymphomas and CNS neoplasms were the most frequently occurring cancers in both males and females, with ASRs of 48.0 and 14.8, 11.4 and 17.0 and 8.7 and 6.9 cases per million in males and females, respectively. Except for neuroblastoma, soft tissue sarcomas, and other and unspecified neoplasms, the incidence rates of all other cancers were higher in males compared with females.

**TABLE 3:** Childhood cancers identified in the database from 2008 to 2017 by malignancy type, gender ratio and gender-specific crude and age-standardised incidence rates.

Malignancy type	Number of cases in gender groups				M/F†	Crude incidence rates‡		ASR§	
	Male		Female			Male	Female	Male	Female
	*n*	%	*n*	%					
Leukaemias	51	29.5	18	10.4	2.8	47.9	17.4	47.5	17.5
Lymphomas	15	8.7	9	5.2	1.7	14.1	8.7	14.2	9.0
CNS neoplasms	12	6.9	7	4.0	1.7	11.3	6.8	11.6	6.6
Neuroblastoma	1	0.6	2	1.2	0.5	0.9	1.9	0.8	2.1
Retinoblastoma	5	2.9	1	0.6	5.0	4.7	1.0	4.6	0.9
Renal tumours	5	2.9	2	1.2	2.5	4.7	1.9	4.3	1.8
Hepatic tumours	1	0.6	0	0.0	0.0	0.9	0.0	0.9	0.0
Bone tumours	11	6.4	1	0.6	11.0	10.3	1.0	11.0	1.1
Soft tissue sarcomas	3	1.7	4	2.3	0.8	2.8	3.9	3.1	3.6
Germ cell tumours	3	1.7	3	1.7	1.0	2.8	2.9	3.1	2.9
Carcinoma and melanomas	11	6.4	6	3.5	1.8	10.3	5.8	10.4	5.5
Other and unspecified neoplasms	1	0.6	1	0.6	1.0	0.9	1.0	0.8	1.1
**Total**	**119**	**68.8**	**54**	**31.2**	**2.2**	**111.6**	**52.4**	**112.3**	**51.9**

ASR, age-standardised incidence rate; CNS, central nervous system.

† , M/F: male-to-female ratio.

‡ , Crude incidence rates expressed per 1 000 000 persons younger than 19 years.

§ , ASR: Age-standardised incidence rates expressed per 1 000 000 persons younger than 19 years.

### Geographical trends in childhood cancer incidence

Using prescriber’s postal codes as a proxy, differences in the incidence of childhood cancers were observed in the various geographical regions on the database. No new cases of cancers in Eastern Cape, Mpumalanga and Northern Cape, based on prescribers’ postal codes, were identified in the database during the study period (Table 1). Leukaemias were the most frequently identified childhood cancers in the Free State, Gauteng, KwaZulu-Natal and the Western Cape. Only one new case of a hepatic tumour was identified during the study period and was recorded in the Western Cape.

Incidence rates (per million persons) were highest in KwaZulu-Natal (193.4 per million persons), followed by Gauteng (102.3 per million persons) and the Western Cape (77.3 per million persons). With the exception of North West and Limpopo, leukaemias were the most frequently identified cancers, with incidence rates ranging from 29.0 to 56.9 cases per million persons, across provinces that recorded new cases of childhood cancers during the study period (Table 4).

**TABLE 4:** Geographical trends in the incidence of childhood cancers over the study period.

Malignancy type	Incidence† in the province (2008–2017)										
	Eastern Cape	Free State	Gauteng	KwaZulu-Natal	Limpopo	Mpumalanga	Northern Cape	North West	Western Cape	Other‡	All
Leukaemias	0.0	38.0	47.6	56.9	0.0	0.0	0.0	0.0	29.0	61.0	32.6
Lymphomas	0.0	0.0	11.9	34.1	8.6	0.0	0.0	17.0	6.5	0.0	11.7
CNS neoplasms	0.0	0.0	7.1	37.9	0.0	0.0	0.0	0.0	9.7	0.0	9.1
Neuroblastoma	0.0	0.0	1.2	0.0	0.0	0.0	0.0	0.0	6.5	0.0	1.4
Retinoblastoma	0.0	9.5	4.8	3.8	0.0	0.0	0.0	0.0	0.0	0.0	2.8
Renal tumours	0.0	0.0	7.1	3.8	0.0	0.0	0.0	0.0	0.0	0.0	3.1
Hepatic tumours	0.0	0.0	0.0	0.0	0.0	0.0	0.0	0.0	3.2	0.0	0.5
Bone tumours	0.0	9.5	4.8	11.4	8.6	0.0	0.0	0.0	9.7	0.0	6.0
Soft tissue sarcomas	0.0	9.5	4.8	0.0	0.0	0.0	0.0	0.0	6.5	0.0	3.3
Germ cell tumours	0.0	9.5	3.6	7.6	0.0	0.0	0.0	0.0	0.0	0.0	2.9
Carcinoma and melanomas	0.0	0.0	7.1	37.9	0.0	0.0	0.0	0.0	3.2	0.0	7.9
Other and unspecified neoplasms	0.0	0.0	2.4	0.0	0.0	0.0	0.0	0.0	0.0	0.0	1.0
**Total**	**0.0**	**76.0**	**102.3**	**193.4**	**17.2**	**0.0**	**0.0**	**17.0**	**74.4**	**61.0**	**82.3**

CNS, central nervous system.

† , Incidence rates expressed per 1 000 000 persons younger than 19 years.

‡ , Case(s) with missing prescriber postal code.

### Time trends in childhood cancer incidence

**FIGURE 1:**
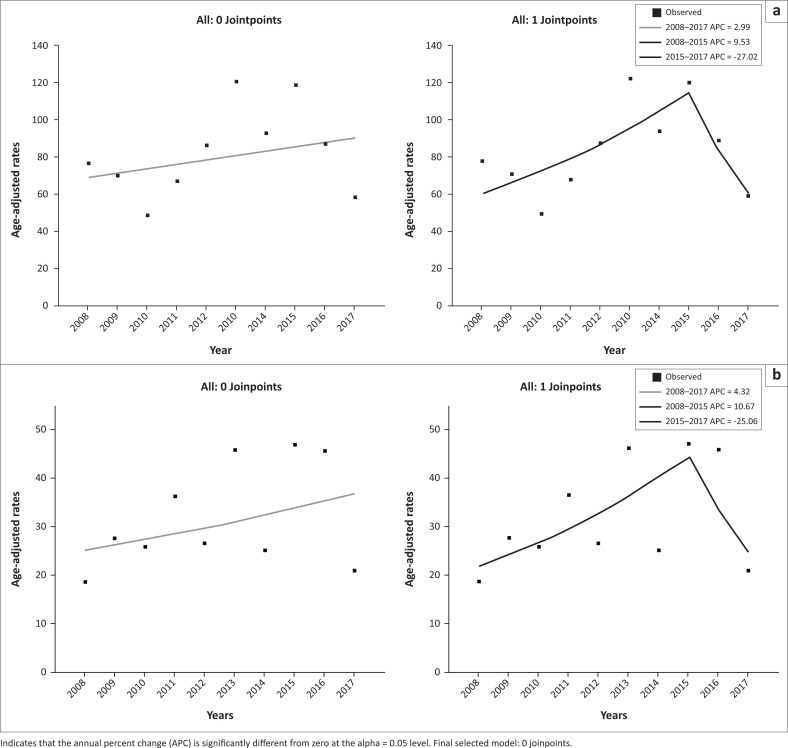
(a) Temporal trends in the incidence of all childhood cancers combined from 2008 to 2017; (b) Temporal trends in incidence of leukaemias from 2008 to 2017.

Table 5 indicates the incidence rates of all childhood cancers from 2008 to 2017. Figure 1a and 1b indicates the trend in ASR of all cancers combined and leukaemia, respectively, with their corresponding APCs. Overall, incidence rates of childhood cancers decreased from 76.7 cases per million persons in 2008 to 58.2 cases per million persons in 2017; however, peaking at 120.3 cases per million persons in 2013. Incidence rates for all cancers combined increased from 2008 to 2015 (APC of 9.53%; 95% CI: –2.9 to 23.6) (*p* = 0.1) and decreased thereafter from 2015 to 2017 (APC of –27%; 95% CI: –70.5 to 80.3) (*p* = 0.4). With the exception of 2008, in which carcinomas and melanomas were the most frequently diagnosed cancer, leukaemias were consistently found to be the most frequently diagnosed cancer, annually, from 2009 to 2017. Temporal trends in the incidence of leukaemias were similar to those of all cancers combined, with an APC of 10.67% (95% CI –2.9 to 26.2) (*p* = 0.1) from 2008 to 2015 and an APC of –25.0% (95% CI –71.9 to 99.8) (*p* = 0.5) from 2015 to 2017.

**TABLE 5:** Age-standardised incidence rates of childhood cancers over the study period.

Malignancy type	Annual age-standardised incidence rates†									
	2008	2009	2010	2011	2012	2013	2014	2015	2016	2017
Leukaemias	18.7	27.7	25.9	36.3	26.6	46.0	25.1	47.0	45.8	21.0
Lymphomas	14.4	7.6	8.9	5.2	5.1	30.1	6.2	22.0	11.9	9.3
CNS neoplasms	0.0	7.6	4.7	4.8	20.8	12.6	4.9	19.9	3.9	11.4
Neuroblastoma	0.0	0.0	0.0	0.0	0.0	0.0	9.2	0.0	4.5	0.0
Retinoblastoma	0.0	3.9	0.0	0.0	0.0	0.0	14.1	0.0	8.4	0.0
Renal tumours	5.0	0.0	0.0	0.0	5.6	4.8	4.4	4.0	0.0	7.1
Hepatic tumours	0.0	0.0	0.0	0.0	0.0	0.0	0.0	0.0	4.3	0.0
Bone tumours	0.0	0.0	4.7	0.0	0.0	16.3	18.5	10.5	8.7	5.4
Soft tissue sarcomas	0.0	4.0	4.3	5.6	10.5	0.0	4.4	4.5	0.0	0.0
Germ cell tumours	4.7	0.0	0.0	9.6	6.3	0.0	6.2	4.5	0.0	0.0
Carcinoma and melanomas	33.9	11.1	0.0	5.2	11.1	10.5	0.0	6.0	0.0	4.2
Other and unspecified neoplasms	0.0	7.9	0.0	0.0	0.0	0.0	0.0	0.0	0.0	0.0
**Total**	**76.7**	**69.8**	**48.6**	**66.9**	**86.2**	**120.3**	**92.7**	**118.3**	**87.6**	**58.2**

CNS, central nervous system.

† , Age-standardised incidence rate expressed per 1 000 000 persons younger than 19 years.

## Discussion

This study reports on the incidence and temporal trends in the incidence of cancers in children aged younger than 19 years in a section of the South African private health sector over 10 years (2008–2017). The overall ASR for all childhood cancers over the study period was 82.3 per million persons, with leukaemias, lymphomas and CNS neoplasms constituting the top three most frequently identified cancers.

Previous epidemiological studies into childhood cancers in South Africa have been carried out in children younger than 15 years using data from South African cancer registries (Erdmann et al. 2015:2631; Stefan et al. 2015:944; Steliarova-Foucher et al. 2017a). These studies by Erdmann et al. (2015) (SACTR data, 1987–2007), Stefan et al. (2015) (NCR data, 2000–2006) and Steliarova-Foucher et al. (2017a) (NCR data, 1998–2012) reported ASRs of 45.7, 45.6 and 45.2 cases per million population, respectively. The estimated ASR for all cancers in children aged younger than 15 years in our study population was, therefore, almost double of those previously reported in South Africa. It should, however, be noted that these studies made use of national cancer registry data (thus included both private and public sector), and that the most recent year under review for the previous studies was 2012 (Steliarova-Foucher et al. 2017a). The higher estimate in our study is, therefore, conceivable considering the increasing incidence of childhood cancers in recent times (Steliarova-Foucher et al. 2017a). The higher observed ASR (73.4 per million) in our study can also be ascribed to the composition of the study population – patients subscribed to medical schemes are more likely to have access to modern diagnostic procedures and, therefore, more incident cases of cancers, when present, will be identified. It is, however, still far lower than the ASR of nearly 180 per million observed in developed countries such as the United States of America (Ward et al. 2014).

The 5–9 year age group had the highest age-specific incidence rate (112.1 per million) among children aged 0–14 years. This is in contrast to the highest age-specific incidence rate in the under-5 years’ age group in previous studies conducted using national data in South Africa (Erdmann et al. 2015:2631; Stefan et al. 2015:942; Steliarova-Foucher et al. 2017a), as well as most countries around the globe (Bidwell et al. 2019; Erdmann et al. 2018:24; Steliarova-Foucher 2017b:722).

The observed male preponderance in the incidence of childhood cancers in this study accords with most studies conducted in different parts of the globe which have reported a higher incidence of childhood cancers in males than females (Dorak & Karpuzoglu 2012; Siegel et al. 2018, Spector, Pankratz & Marcotte 2015:10). This trend has also been demonstrated in previous studies conducted in South Africa, although relatively lower M/F ratio of between 1.2 and 1.3 was reported in these studies (Erdmann et al. 2015:2631; Stefan et al. 2015:944; Steliarova-Foucher et al. 2017a).

Leukaemias were observed to be the most frequently diagnosed childhood cancer across all age-groups and in both males and females in this study, and this is similar to the results of previous studies in South Africa (Erdmann et al. 2015:2633; Stefan et al. 2015:944; Steliarova-Foucher et al. 2017a) This is, however, in contrast to the higher incidence of lymphomas, compared to leukaemias, in other countries in sub-Saharan Africa (Stefan 2015b). Lymphomas are usually the most frequently occurring cancers in adolescents (Steliarova-Foucher et al. 2017b:727). The higher incidence of leukaemias, in comparison to lymphomas, in the adolescent age group in this study confirms a predominance of leukaemias among childhood cancers in our patient population. Our findings, which show the highest prevalence of leukaemia, followed by lymphomas and CNS neoplasms among other childhood cancers, are in line with international trends which indicate leukaemias, lymphomas and CNS neoplasms as the top three most common childhood cancers (Bidwell et al. 2019; Erdmann et al. 2018; Moreno et al. 2013).

Overall, there was a 24.1% decrease in the incidence rate of all childhood cancers combined from 2008 to 2017, although the decrease was not homogenous throughout the study period. This is in contrast to the increasing trend in incidence of childhood cancers observed by Steliarova-Foucher et al. (2017b:727) in their study using data from 153 registries from 62 countries around the globe. The database used for this study contains medicine claims of patients subscribed to medical aid schemes whose medicine benefits are managed by the PBM company. Composition of the database is, consequently, likely to change annually and this could explain the fluctuations in the incidence of cancers observed in this study. Furthermore, annual changes in the health plan or benefit design of beneficiaries could account for the annual fluctuations in incidence rates observed in this study, as benefit design defines the composition of a population at a given time (Rassen et al. 2019). This information, however, was not available for analysis, and could therefore not be controlled for. An interesting observation is the highest incidence of carcinomas and melanomas, among all other diagnostic groups in 2008 (33.9 cases per million). Mandatory coding of diseases for reimbursement purposes using ICD-10 codes started in 2008 in South Africa; this observation may, therefore, reflect less precision in the coding processes in 2008 and not necessarily a high incidence of this diagnostic group because carcinomas in children are relatively rare (Elhassan et al. 2018). This was confirmed by a reduction in the incidence of carcinomas and melanomas in subsequent years.

Based on prescribers’ postal codes, incidence rates of children and adolescents receiving treatment for cancers were found to be highest in KwaZulu-Natal followed by Gauteng and Western Cape, although the largest proportion of incident cases was identified in Gauteng. This is in contrast to a previous study in South Africa that reported the highest ASR of childhood cancer in Western Cape (Stefan et al. 2015:941). Treatment of childhood cancers in South Africa is carried out in specialised centres which are mostly located in the major metropolitan municipalities. This implies that patients may receive cancer treatment at locations other than their province of residence. The leukaemias were the diagnostic group with the highest incidence rates in four out of the six provinces which recorded cases of childhood cancers, further confirming leukaemias as the most prevalent cancer in the section of the private health sector studied.

## Strengths and limitations

The study population was drawn from the database of only one PBM company in South Africa. The external validity of this study is therefore limited because results cannot be generalised to the total private health sector or South African population as a whole. Further research using up-to-date population-based data may have the potential of confirming these findings and describing the general trends in the whole South African population. Although higher incidence rates were recorded in this study, under-ascertainment of cases cannot be ruled out because data used were reimbursed claims. Patients diagnosed with cancers that are not captured in their prescribed minimum benefits, and who do not have comprehensive health plans with oncology benefits would have to make out-of-pocket payments for cytotoxic medication. Data on these patients will, therefore, not be included in the database. The use of prescribers’ postal codes as a proxy for the geographic location of patients has the potential of introducing bias into the analysis of geographical trends. This is because most oncologists have their practices located in metropolitan municipalities and, therefore, patients may travel to those provinces for treatment. Higher incidence rates in those provinces may, therefore, reflect a higher diagnostic rate of cancers and not necessarily the true incidence of cancers.

Notwithstanding the limitations of this study, results obtained provide baseline information on the current trends of childhood cancers in a section of the private health sector of South Africa and have the potential to stimulate further research into childhood cancers in the whole South African population.

## Conclusion

The findings of this research provide an insight into the epidemiological trends of cancers in children and adolescents in a section of the South African private health sector. The highest incidence rate of cancers was estimated for the adolescent age group. The overall incidence rate observed in this study was largely driven by leukaemias which contributed more than one-third of the cases identified on the database. Results of the temporal trend analysis do not support the observed increasing rates of childhood cancers in global communities. Data used for this study was, however, obtained from only one database. Future studies using a larger and more nationally representative database should be carried out to establish the true epidemiological trends in childhood cancers in South Africa.
